# Autopsy and statistical evidence of disturbed hemostasis progress in COVID-19: medical records from 407 patients

**DOI:** 10.1186/s12959-020-00256-5

**Published:** 2021-02-10

**Authors:** Tiebin Jiang, Bo Lv, Hongxia Liu, Shiwen He, Guogang Zhang, Chanyi Li, Wanqiong Li, Weilin Li, Yaqi He, Tong Zhang, Yunyun Wang, Wu Mo, Ning Yi, Luying Peng, Ying Li, Chunhong Ruan, Chengyuan Li, Yaqi Liu, Peipei Luo, Huan Jiang, Zhigang Xue, Liang Liu, Wenjun Wang

**Affiliations:** 1 Department of Hematology, The Third Xiangya Hospital, Central South University, Changsha, 410013 Hunan China; 2 Department of Regenerative Medicine, School of Medicine, Tongji University, 1239 Siping Road, Shanghai, 200092 China; 3 Scientometrics and Evaluation Center for Rule of Law, China University of Political Science and Law, Beijing, 100088 China; 4 School of Computer Science and Engineering, Central South University, Changsha, 410083 China; 5 Department of Cardiovascular, The Third Xiangya Hospital, The Central South University, Changsha, 410013 Hunan China; 6 Reproductive Medicine Center, Tongji Hospital, Tongji University, Shanghai, 200065 China; 7 Stem Cell and Regenerative Medicine Engineering Research Center of Hunan Province, Hunan Yuanpin Cell Technology Co. Ltd, 102 Dongwu Road, Changsha City, 410100 Hunan Province China; 8 Department of Forensic Medicine, Tongji Medical College of Huazhong University of Science and Technology, 13 Hangkong Road, Wuhan City, 430030 Hubei Province China; 9 Department of Spinal Surgery, The First Affiliated Hospital of University of South China, Hengyang, 421002 Hunan China

**Keywords:** COVID-19, Thrombosis, Hemostasis, Autopsy, Statistics

## Abstract

**Background:**

The progression of coagulation in COVID-19 patients with confirmed discharge status and the combination of autopsy with complete hemostasis parameters have not been well studied.

**Objective:**

To clarify the thrombotic phenomena and hemostasis state in COVID-19 patients based on epidemiological statistics combining autopsy and statistical analysis.

**Methods:**

Using autopsy results from 9 patients with COVID-19 pneumonia and the medical records of 407 patients, including 39 deceased patients whose discharge status was certain, time-sequential changes in 11 relevant indices within mild, severe and critical infection throughout hospitalization according to the Chinese National Health Commission (NHC) guidelines were evaluated. Statistical tools were applied to calculate the importance of 11 indices and the correlation between those indices and the severity of COVID-19.

**Results:**

At the beginning of hospitalization, platelet (PLT) counts were significantly reduced in critically ill patients compared with severely or mildly ill patients. Blood glucose (GLU), prothrombin time (PT), activated partial thromboplastin time (APTT), and D-dimer levels in critical patients were increased compared with mild and severe patients during the entire admission period. The International Society on Thrombosis and Haemostasis (ISTH) disseminated intravascular coagulation (DIC) score was also high in critical patients. In the relatively late stage of nonsurvivors, the temporal changes in PLT count, PT, and D-dimer levels were significantly different from those in survivors. A random forest model indicated that the most important feature was PT followed by D-dimer, indicating their positive associations with disease severity. Autopsy of deceased patients fulfilling diagnostic criteria for DIC revealed microthromboses in multiple organs.

**Conclusions:**

Combining autopsy data, time-sequential changes and statistical methods to explore hemostasis-relevant indices among the different severities of the disease helps guide therapy and detect prognosis in COVID-19 infection.

**Supplementary Information:**

The online version contains supplementary material available at 10.1186/s12959-020-00256-5.

## Essentials

The role of hemostasis-related indices in different severities of COVID-19 remains to be clarified.

Autopsy, coagulation indices after admission or before outcome, and statistical analysis were used to examine the hemostatic process and the importance of related parameters.

Persistent abnormalities in coagulation parameters during hospitalization indicated poor prognosis.

Prothrombin time, D-dimer levels, blood glucose levels, and age are the most important parameters related to the severity of COVID-19.

## Introduction

Severe acute respiratory syndrome coronavirus 2 (SARS-CoV-2) was first identified in Wuhan, China and was associated with an outbreak of coronavirus disease 2019 (COVID-19) that affected more than 9 million patients with more than 480 thousand deaths globally as of July 18th, 2020 [1–4]. The coagulation changes in SARS-CoV-2-infected patients are now described as COVID-19-associated coagulopathy (CAC) [5–8]. A few clinical reports have been published on patients with COVID-19 who undergo complete autopsy [9–17]. Notably, in several cases, a high incidence of venous thromboses and embolisms are noted as the direct cause of death [18–20]. CAC-correlated thrombotic phenomena in COVID-19 deaths have been suggested to have significant poor prognostic features [12, 21–24]. However, the progression of coagulation in COVID-19 patients with confirmed discharge status (death or discharge) and the combination of autopsy with complete coagulation parameters have not been well studied.

Here, we report the pathological features of COVID-19 patients with thrombotic phenomena from autopsy and coagulation disease progression among mild, severe, and critical patients from one hospital in Wuhan, China. Moreover, we also investigated the difference between survivors and nonsurvivors in patients with critical infection. Finally, we evaluated the correlation and contribution of those features regarding the severity of patients using informatic tools.

## Methods

### Patients

We collected autopsy data from 9 deceased patients and clinical data from 407 patients in one hospital in Wuhan. All patients were confirmed to have COVID-19 pneumonia according to the Chinese National Health Commission (NHC) guidelines (7th trial edition). Briefly, patients who were SARS-CoV-2 positive using RT-PCR were further confirmed to have pneumonia using computed tomography (CT) scanning. Patients’ medical records contain the essential information and values of detection indices during the whole process from admission to discharge (or death).

### Autopsy and histological examination

We performed full-body autopsies on 9 deceased persons with SARS–CoV-2 positivity as soon as possible after taking proper safety precautions at biosafety level 3 (BSL-3) following guidelines from the industrial standards of public safety of the People’s Republic of China. Tissue samples for histopathologic examination were fixed in buffered 4% formaldehyde and processed via a standard procedure to stain slides with hematoxylin–eosin (H&E). All hematological indices were collected for the last testing before death.

### Data collection and procedures

We reviewed the electronic clinical charts, examination records, and laboratory findings for 407 COVID-19 patients (including 39 deceased patients). During the whole process from admission to discharge (or death), time-sequential investigations including 11 indices, six of which were hemostasis-related indices detected by the kits from Siemens (German) using Sysmex 5100 (Japan) (platelet (PLT), prothrombin time (PT), activated partial thromboplastin time (APTT), thrombin time (TT), fibrinogen (FIB), D-dimer (latex immunoturbidimetry)) and five of which were lab parameters detected by the kits from Leadman (China) using Hitachi Automatic Analyzer 7600 (Japan) (blood glucose (GLU), total cholesterol (TC), triglyceride (TG), high-density lipoprotein (HDL) and low-density lipoprotein (LDL)) were extracted for subsequent analyses [25]. Other clinical characteristics, including age, sex, major comorbidities (coronary artery disease [CAD], hypertension [HTN], and diabetes mellitus [DM]), discharge status (survival or death) and hospitalization time, were also analyzed in our study. Following data extraction, those patients were divided into 3 groups (mild, severe or critical) according to the NHC guidelines for COVID-19 pneumonia [26]. Furthermore, we focused on the temporal changes in these indices between 39 nonsurvivors and 42 survivors in the critical group to assess the coagulation process of disease deterioration. In addition, correlation using the Spearman method and a random forest model was calculated for patient classification to evaluate the importance of 11 indicators for the process of COVID-19. For DIC analysis, the International Society on Thrombosis and Haemostasis (ISTH) diagnostic criteria were applied to all the patients [5, 27].

### Case definitions

Mild infection, severe infection, and critical infection were characterized throughout the entire hospitalization according to NHC guidelines [26]. Briefly, mild infection is characterized by mild symptoms, fever, respiratory symptoms, and imaging findings of pneumonia. Severe infection is noted if any of the following appears: shortness of breath (RR > 30 times/min), oxygen saturation ≤ 93%, and PaO_2_/FiO_2_ ≤ 300 mmHg. Critical infection is characterized by any of the following appearances: respiratory failure requires mechanical ventilation, shock, and other organ failure requires ICU monitoring treatment.

### Spearman correlation coefficient and random Forest model analysis

We labeled t male as 1 and female as 0 in the random forest model. Due to the limitations of our detection system, the reportable ranges of D-dimer and TT were 0.22–21 μg/mL and 13–240 s, respectively. Therefore, when the level was beyond this range (e.g., > 21 μg/mL for D-dimer), we corrected those values to the barrier of the reportable range (e.g., > 21 μg/mL for D-dimer as 21 μg/mL). We also labeled the severity of patients as 1 for mild syndrome, 2 for severe syndrome, and 3 for critical syndrome in the correlation coefficient analysis [28]. Then, all data were put into one file to calculate the Spearman correlation coefficient (R, 3.6.1, package ‘PerformanceAnalytics’) and random forest model (Python 3.7) according to previous reports [29, 30]. Briefly, we used the severity of patients as 1 for mild syndrome, 2 for severe syndrome, and 3 for critical syndrome as the outcome variable for the random forest model. When constructing the model, the decision tree is enerated by the CART algorithm using the Gini index (also identified as the importance index in our study). The Gini index represents the impurity level of the model. A lower Gini index indicates lower purity. In the classification problem, assuming there are K categories, the probability of the kth category is pk, and the Gini index formula is as follows:

$$ \mathrm{Gini}\left(\mathrm{D}\right)=\sum \limits_{\mathrm{k}\hbox{-} 1}^{\mathrm{K}}{\mathrm{p}}_{\mathrm{k}}\left(1\hbox{-} {\mathrm{p}}_{\mathrm{k}}\right)=1\hbox{-} \sum \limits_{\mathrm{k}\hbox{-} 1}^{\mathrm{K}}{\mathrm{p}}_{\mathrm{k}}^2 $$

We then used the Gini index gain as the basis for selecting the features of the decision tree using the following formula:

$$ \Delta \mathrm{Gini}\left(\mathrm{A}\right)=\mathrm{Gini}\left(\mathrm{D}\right)\hbox{-} {\mathrm{Gini}}_{\wedge}\left(\mathrm{D}\right) $$

We choose the maximum value of the Gini index gain as the splitting characteristic, and the node is used as the split condition.

### Statistical analysis

Descriptive analyses of categorical variables and baseline indices were expressed as medians [interquartile ranges (IQRs)] or numbers (%). Mean and standard error were also used to display the line charts of index changes. Proportions for categorical variables were compared using the χ^2^ test. Continuous variables were compared using the Wilcoxon rank sum test. These statistical analyses were performed using R (version 3.6.1), and graphs were drawn using GraphPad Prism (version 8.0.2).

### Role of funding source

None of the funders had any role in the study design, data collection, analysis, interpretation or in the writing of the article or the decision to submit it for publication. Independence from funders and sponsors was confirmed by the researchers.

## Results

Complete autopsies of 9 deceased COVID-19 patients (5 males and 4 females) with 15 median hospitalization days (IQR, 10–22) before death were performed (Table 1). The median of ages was 67 years old (IQR, 63–78). Except for the missing comorbidity records of 2 cases (cases 6 and 9), the other 7 cases had comorbidities. Specifically, 7/7 patients had hypertension, 2/7 (cases 1 and 4) had cerebral infarction, 2/7 (cases 5 and 8) had coronary artery disease, and 1/7 (case 7) had gout. Of note, one case (case 5) had hypertension, coronary artery disease, renal dysfunction, lacunar infarction, and chronic bronchitis with emphysema. A total of 8/9 patients died mainly due to respiratory failure with multiple organ failure, and the other 1/9 died due to sudden cardiac death from acute coronary heart disease.

**Table 1:** Demographic and clinical characteristics and laboratory indices of 9 autopsy cases

Case	1	2	3	4	5	6	7	8	9	Median (IQR)
Age - years	70	66	53	83	80	62	51	78	67	67 (63–78)
Gender	Male	Female	Female	Male	Female	Male	Male	Female	Male	
Hospital stay - days	20	10	22	29	5	15	5	12	22	15 (10–22)
Comorbidities	Cerebral infarction, hypertension	Hypertension	Hypertension	Cerebral infarction, hypertension	Hypertension, coronary heart disease, renal dysfunction, lacunar infarction, slow to emphysema	NA	Hypertension (level 3 with very high risk), gout	Hypertension, coronary artery disease	NA	
**Hyaline thrombus distribution of major organs**^**a**^										
**Lung**	**√**	**√**	**√**	**√**	**√**	**√**	**√**	**√**	**√**	
**Kidney**		**√**							**√**	
**Heart**				**√**						
**Brain**	**√**	**√**	**√**		**√**					
**Spleen**	**√**	**√**	**√**		**√**					
**Laboratory findings of coagulation relevant indices**^**b**^										
**PLT - × 10**^**9**^**/L**	63	127	116	134	76	167	161	66	174	161.0 (134.0–167.0)
**PT - s**	19.2	13.5	13	12	14.5	13.7	126.2	19.3	12	12.5 (12.0–13.1)
**APTT - s**	45.2	27.9	23.4	35.1	46.3	41.9	83.5	41.6	35.1	31.5 (26.8–35.1)
**TT - s**	65.2	14.9	16.7	16.2	18.2	16.7	27.8	NA	16.2	16.5 (16.2–16.7)
**FIB - g/L**	12.73	45.43	18.76	3.36	1.3	1.3	15.94	77.33	3.36	1.3 (1.3–2.3)
**D-dimer - μg/mL**	1.5	6.2	2.2	3.6	6.1	3.6	3.6	NA	NA	3.6 (3.3–3.6)
**DIC**	6	5	5	5	5	4	5	6	5	5.0 (5.0–5.0)

^a^ Blank cells represent no obvious thrombi in those organs. ^b^ NA indicate the data are not available

**Fig. 1:**
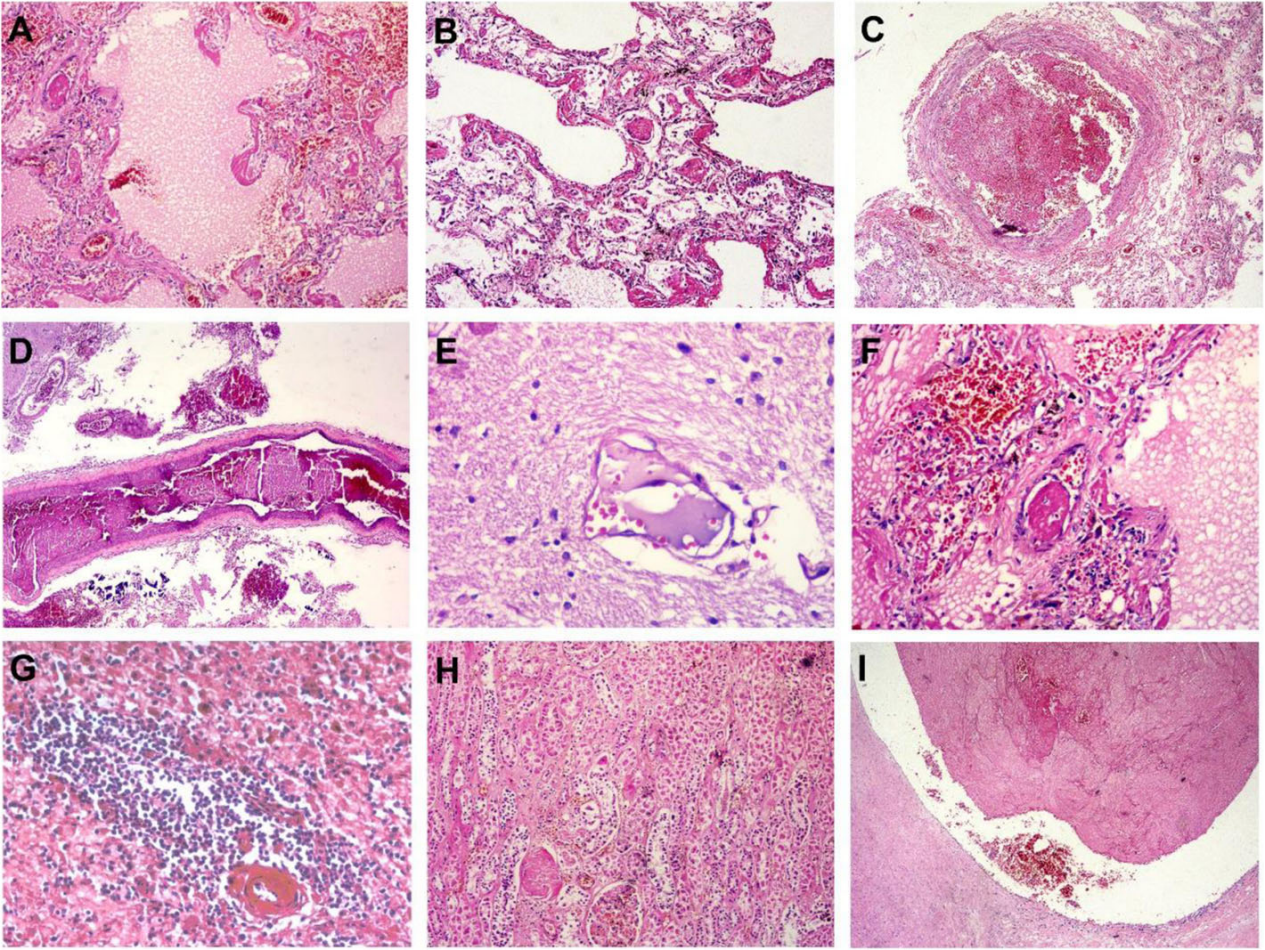
Histopathological findings of deaths associated with COVID-19 (**h**&**e** staining, original magnification). **a**-**c**, thrombi in the lung: small intravascular microthrombus (**a**) (case 1; 100×), alveolar capillary and interstitial vascular microthrombi (**b**) (case 4; 40×), microthrombus of right hilar arteriole (**c**) (case 4; 25×); **d**-**e**, thrombi in the brain: thrombosis of the subarachnoid arteriole in the right parietal lobe (**d**) (case 1; 40×), microthrombus was observed in the lumen of small vessels in parenchyma (**e**) (case 3; 40×); **f**-**g**, thrombi in the spleen: small intravascular microthrombus (**f**: case 2; 200×; **g**: case 9; 200×); **h**, thrombi in the kidney (case 2; 100×); **i**, coronary artery lumen together with hemorrhage (case 4; 25×)

In addition to diffuse alveolar damage in the lung, the predominant histological findings included hyaline thrombi among all 9 deceased patients (Fig. 1). Specifically, 9/9 cases exhibited microthrombi in the hilar arteriole, alveolar wall capillary and interstitial vascular lumen of the lung; 4/9 (1, 2, 3, and 5) in the subarachnoid arteriole and parenchymal small endovascular lumen of the brain; 4/9 (1, 2, 3, and 5) in the small vascular lumen of the spleen; 2/9 (cases 2 and 9) within the kidney; and 1/9 (case 4) in the coronary artery lumen together with hemorrhage. To evaluate the coagulation state before death, we also extracted the last hematological indices relevant to hemostasis, i.e., platelet, prothrombin time, activated partial thromboplastin time, thrombin time, fibrinogen, and D-dimer in these cases when they were alive (Table 1). The ISTH DIC scores in 8/9 cases matched the diagnostic criteria for DIC (≥5 points).

The autopsy results of thrombi in the major organs of the body and their DIC scores before death strongly indicated coagulation abnormalities in COVID-19 patients (Table 1**,** Fig. 1). Together with previous reports demonstrating the high relevance of blood glucose, total cholesterol, triglycerides, high-density lipoprotein, and low-density lipoprotein with coagulation [31–34], we then included those indices in our clinical data analyses. The clinical time-sequential data included 407 hospitalized patients with confirmed COVID-19. Their demographic and clinical characteristics are shown in Table 2. The median (IQR) age was 62.0 years (51.0–58.0) with an overall range from 6 years to 92 years; 51.8% of the patients were from 40 to 65 years of age. A total of 49.9% were female. Of all the patients, 184 (45.2%) had at least 1 of the following 3 comorbidities: coronary artery disease (40 [9.8%]), hypertension (144 [35.4%]), and diabetes (72 [17.7%]). Since all the patients included in this study had either been discharged from the hospital with SARS-CoV-2 negativity (368 [90.4%]) or died (39 [9.58%]), the median duration of hospitalization was 15 days (8.0–25.0).

**Table 2:** Demographic and clinical characteristics of 407 patients with COVID-19

Characteristics	All Patients (***n*** = 407)	Disease Severity		
		Mild (***n*** = 253)	Severe (***n*** = 73)	Critical (***n*** = 81)
Age - Median (IQR) - years	62.0 (51.0–69.0)	59.0 (45.0–66.0)	67.0 (60.0–73.0)	65.0 (57.0–72.0)
Distribution - No. (%)				
<40	43 (10.6)	36 (14.2)	4 (5.5)	3 (3.7)
40–65	211 (51.8)	150 (59.3)	22 (30.1)	39 (48.1)
>65	153 (37.6)	67 (26.5)	47 (64.4)	39 (48.1)
Range	6–92	6–92	32–91	24–92
Gender – No. (%)				
Female	203 (49.9)	142 (56.1)	29 (39.7)	32 (39.5)
Male	204 (50.1)	111 (43.9)	44 (60.3)	49 (60.5)
Major Comorbidities – No. (%)	184 (45.2)	98 (24.1)	47 (64.4)	39 (48.1)
Coronary artery disease	40 (9.8)	17 (6.7)	11 (15.1)	12 (14.8)
Hypertension	144 (35.4)	77 (30.4)	36 (49.3)	31 (38.3)
Diabetes mellitus	72 (17.7)	43 (17.0)	14 (19.2)	15 (18.5)
Clinical outcome – No. (%)				
Discharged	368 (90.4)	253 (100)	73 (100)	42 (51.9)
Died	39 (9.58)	0	0	39 (48.1)
Hospital stay - Median (IQR) - days	15.0 (8.0–25.0)	13.0 (7.0–22.0)	21.0 (14.0–26.0)	19.0 (7.0–35.0)

Percentages may not total 100 due to rounding

Abbreviations: COVID-19, coronavirus disease 2019; IQR, interquartile range

Among all 407 patients with different symptoms throughout the hospital stay (the other 208 patients were excluded since they were still in the hospital on March 26), 253 patients (62.2%) exhibited mild infection, 73 patients (17.9%) showed severe infection, and 81 patients (19.9%) showed critical infection (Fig. S1). All relevant clinical records were reviewed to classify the patients into critical, severe, or mild groups according to the Chinese National Health Commission (NHC) guidelines (7th trial edition) for COVID-19 pneumonia [26]. The patients with critical or severe infection were significantly older than those with mild infection (median [IQR] age, 65.0 [57.0–72.0] or 67.0 [60.0–73.0] years vs 59.2 [45.0–59.0] years; both *P* values < 0.0001) and more likely to stay longer at hospital after admission (median 19.0 days [7.0–35.0] or 21.0 [14.0–26.0] vs 13.0 [8.0–22.0]; both P values < 0.001), whereas there was no difference between severe and critical cases (*P* = 0.5996 for age and *P* = 0.2806 for hospital stay). Moreover, compared to mildly infected patients, patients with severe infection were more likely to have other underlying comorbidities (47 [64.4%] vs 98 [24.1%]; *P* = 0.0001), especially hypertension (36 [49.3%] vs 77 [30.4%]; *P* = 0.0028) (Table 2, Table S1).

However, 81 critical patients exhibited slightly similar underlying comorbidities as mild patients (39 [48.1%] in critical patients, *P* = 0.1339), and no significant difference in hypertension proportion was noted between these patients (31 [38.3%] in critical patients, *P* = 0.1894) (Table S1). Furthermore, despite no statistically significant difference (*P* = 0.2806), the median hospitalization days of critical patients were slightly reduced compared with those of severe patients (19.0 [7.0–35.0] vs. 21.0 [14.0–26.0]). This led us to think about whether the nonsurvivors displayed any differences in the critical group.

Table S2 demonstrates that when compared with the survivors (42 (51.9%) patients in the critical group) in the critical group, fewer nonsurvivors had underlying comorbidities (13 [33.3%] vs 26 [61.9%]; *P* = 0.01), such as hypertension (8 [20.5%] vs 23 [54.8%]; *P* = 0.002) and diabetes mellitus (3 [7.7%] vs 12 [28.6%]; *P* = 0.02). Thirty-nine nonsurvivors also had shorter hospital stays after admission compared with 42 survivors (median 10.0 days [6.5–16.5] vs 35.0 [21.3–40.5]; *P* < 0.0001). In addition, the percentages of comorbidities and hospital duration in survivors were more similar to those in the severe group compared with those in nonsurvivors from the critical group (Table 2).

**Fig. 2:**
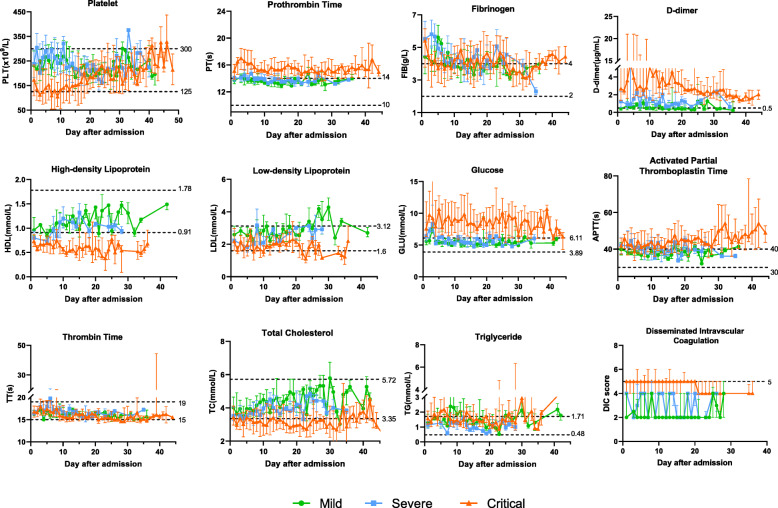
Temporal changes in laboratory indices after admission to discharge/death in patients with COVID-19. Timeline charts illustrate the daily changes in laboratory indices in 407 patients with COVID-19 (253 mild patients, 73 severe patients and 81 critical patients) during hospitalization. The error bars indicate the range of IQRs. The horizontal dotted lines display the upper or lower normal limits of platelet (PLT), prothrombin time (PT), activated partial thromboplastin time (APTT), thrombin time (TT), fibrinogen (FIB), D-dimer, blood glucose (GLU), total cholesterol (TC), triglyceride (TG), high-density lipoprotein (HDL), low-density lipoprotein (LDL), and disseminated intravascular coagulation (DIC) score. Orange triangles, blue squares and green dots indicate critical patients, severe patients and mild patients, respectively. Sample sizes less than 3 were excluded due to statistical significance in the analysis

To determine the major hematological features that appeared during COVID-19 thrombogenic progression, the temporal changes in 11 clinical laboratory indices, including platelet (PLT), prothrombin time (PT), activated partial thromboplastin time (APTT), thrombin time (TT), fibrinogen (FIB), D-dimer, blood glucose (GLU), total cholesterol (TC), triglyceride (TG), high-density lipoprotein (HDL) and low-density lipoprotein (LDL), were tracked on admission until outcome (Table S3). All 407 patients with definite discharge status were analyzed and displayed using a line chart (Fig. 2). During hospitalization, most patients had increased D-dimer levels, and those with critical infection continued to exhibit significantly higher D-dimer levels after admission until the outcome. Intriguingly, PLT in the critical patients exhibited a marked decrease on admission, and the counts remained low until day 14 (both *P* < 0.05 compared to mild and severe patients (Table S3)) and then gradually increased. In addition, the indices, e.g., PT and GLU, in critical patients exhibited persistent prolonged time, higher score or higher level during the hospitalization compared with those in severe or mild patients, whereas other indices, e.g., TC and HDL, in the critical patients were lower at the initial stage and remained at a relatively low level until the outcome. On the other hand, indices, such as LDL, exhibited changes at the late stage, and TT was intermittently prolonged after admission in the critical patients. In contrast, APTT, FIB, and TG exhibited no discernable difference among patients with different levels of severity during hospitalization. We also found persistently high DIC scores in critical patients during hospitalization using the ISTH DIC scoring system.

To evaluate the severity of the coagulation state in patients with different disease levels, we also evaluated the percentages of the peak values of 11 indices in every individual who exhibited a value beyond the normal range during hospitalization. Table S4 and Table S5 summarize the median (IQR) of the level of all the maximum values of 11 indices in every individual patient during hospitalization, the proportions of out-of-normal-range values of all the patients with different levels of severity and their statistical test results. Consistent with previous findings [4, 35], the median concentrations of D-dimer (μg/mL) in all types were greater than the normal range (all patients, 1.15 [0.39–3.18]; normal range, 0–0.5). Nonetheless, critical patients exhibited significantly higher D-dimer levels than those with the other two types (critical median [IQR] vs severe median [IQR] or mild median [IQR]; 16.77 [3.21–12.9] vs 1.60 [0.78–2.94] or 0.53 [0.28–1.39]; two P both < 0.0001). Other coagulation parameters, i.e., PT (17.9 s [15.6–21.9] vs 14.3 s [13.9–14.8] or 14.0 s [13.4–14.5]; two P both < 0.0001), APTT (52.9 s [44.1–68.7] vs 43.6 s [39.9–47.9] or 40.5 s [37.8–44.0]; two P both < 0.0001) and TT (20.0 s [17.3–27.0] vs 17.4 s [16.8–17.7] or vs 16.8 [16.1–17.8]; two P both < 0.0001) were also prolonged in this peak value evaluation. Consistently, the out-of-normal-range portions of those coagulation parameters in critical patients were significantly greater than those in severe or mild patients, e.g., D-dimer (79/80 [98.8%] vs 64/72 [88.9%], *P* = 0.0101; or vs 127/247 [51.4%], *P* < 0.0001), which is similar to that noted in the DIC grades.

**Fig. 3:**
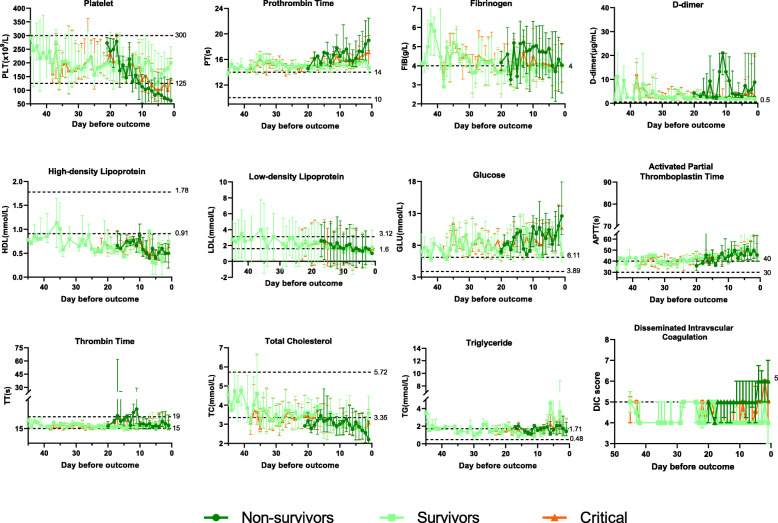
Temporal changes in laboratory indices, including coagulation-relevant parameters, including platelet (PLT), prothrombin time (PT), activated partial thromboplastin time (APTT), thrombin time (TT), fibrinogen (FIB), D-dimer, blood glucose (GLU), total cholesterol (TC), triglyceride (TG), high-density lipoprotein (HDL), low-density lipoprotein (LDL), and disseminated intravascular coagulation (DIC) scores, in critical patients with COVID-19. Timeline charts illustrate the daily changes in parameters in 81 critical patients (orange line with triangles, 39 nonsurvivors and 42 survivors) before discharge (light green line with squares) or death (dark green lines with dots). Sample sizes less than 3 were excluded due to statistical significance in the analysis. The error bars indicate the range of IQRs

High variance of hospital stay and other hematological indices in those 81 critical patients strongly suggested that differences existed between survivors and nonsurvivors (Table 2 and Table S4), so progression analyses of laboratory hematological indices were also performed to evaluate the severity of coagulation. Since all patients were confirmed to be either discharged with SARS-CoV-2 negativity or deceased, we defined the date of discharge or death as day 1 before the outcome, and the previous dates increased backward (Fig. 3, Table S6). Interestingly, most indices of survivors and nonsurvivors exhibited similar trends, medians, and portions of abnormal values for all critical patients. However, when dividing the critical patients into survivors and nonsurvivors, several indices exhibited significant differences between these groups. Combining analysis with the maximum values of individual patients, nonsurvivors presented with fewer platelets (× 10^9^/L)(216.0 [142.5–273.5] vs 287.5 [199.0–370.0]; *P* = 0.0035), prolonged prothrombin time (20.2 s [18.1–25.3] vs 16.7 s [15.3–18.0]; *P* < 0.0001), elevated D-dimer levels (21.00 μg/mL [13.42–21.00] vs 6.79 μg/mL [2.82–20.61]; *P* = 0.0013) and greater DIC score (6 [5–7] vs 5 [5, 6]; *P* = 0.0002) compared with survivors (Table S7) with striking differences noted close to the destination date (*P* = 0.0027 and *P* = 0.0051 for PLT and PT at day 11 before outcome, respectively; *P* = 0.0063 and *P* = 0.0193 for D-D at day 12 before outcome. Notably, although no obvious changes were noted when dividing all patients into 3 groups (Fig. 2), the subgroup of nonsurvivors manifested a significantly higher level of fibrinogen compared with that of survivors at days 7 and 9 before the outcome (Fig. 3**,** Table S6).

**Fig. 4:**
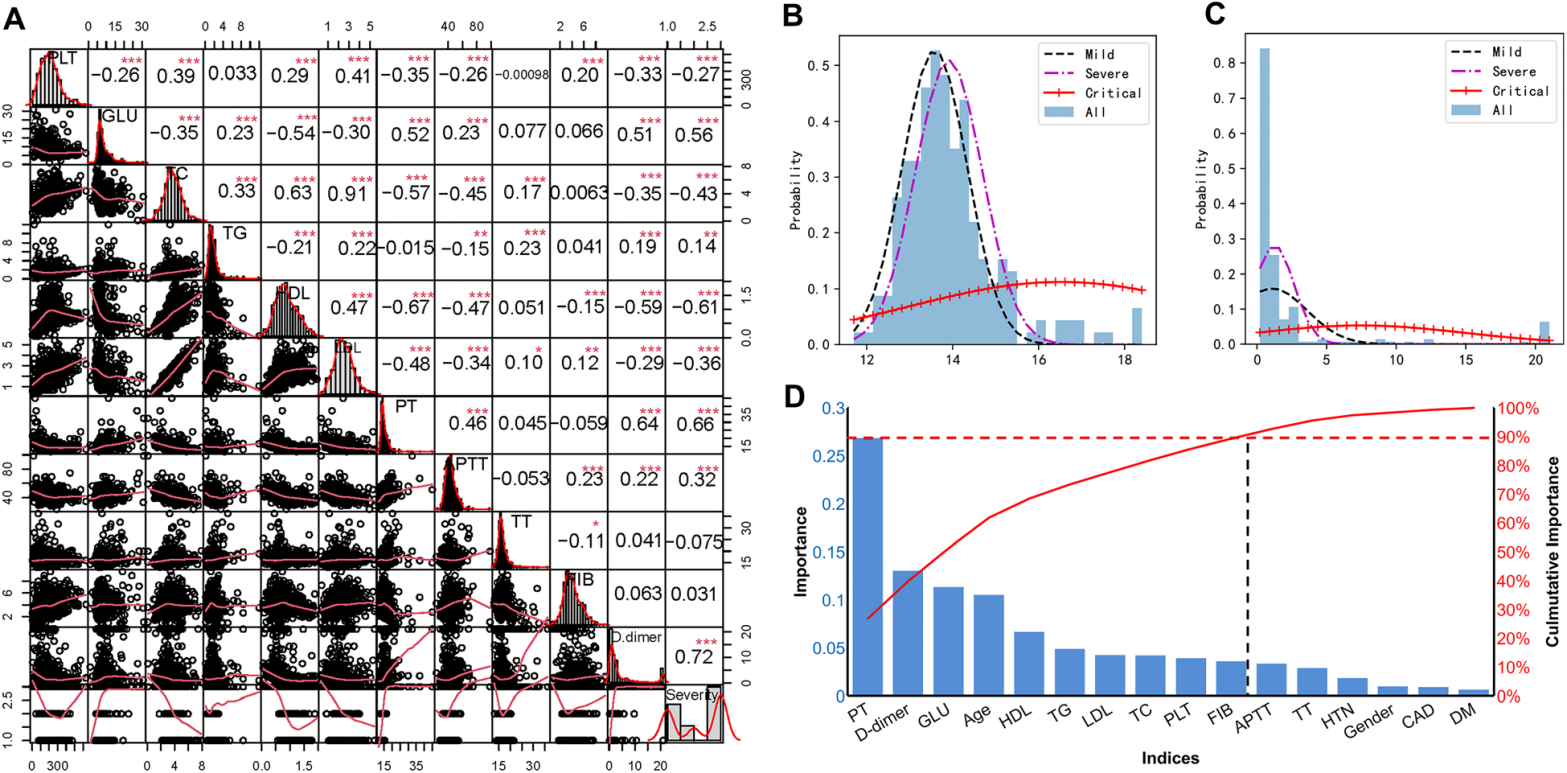
Informatic analysis of mild, severe and critical patients with COVID-19. **a**. Spearman correlation demonstrating the correlation coefficient between different features with three patient categories. Feature ‘Severity’ indicated three patient categories. The maximum values from each individual during the entire hospitalization were used in this analysis. The number in the upper half indicates the correlation value between two indices. The diagonal histograms indicate the distribution of each index. The lower half shows the scatter distribution between two indices. **b**-**c**. Histograms demonstrating the distribution of PT (**b**) and D-dimer (**c**) within three patient categories. The abscissa represents the current feature value, and the ordinate represents the probability with the current feature value. The blue bar is the histogram of all samples, indicating the distribution of data, where the three fitting curves are black, purple, and red, corresponding to the three patient categories, mild, severe, and critical, respectively. **d**. Importance histogram of random forest model results with dotted lines indicating the 90% importance of the model. The blue bars indicate the rate of importance, the red curve indicates the cumulative importance, and the black dashed line divides the top indices when the cumulative importance reaches 90% (red dashed line). *, **, and *** indicate *P* < 0.05, *P* < 0.01, and *P* < 0.001, respectively, for Spearman correlation significance

To further explore the underlying correlation between these groups, a heat map was applied to visualize the Spearman correlation coefficient between each clinical feature or laboratory index (Fig. 4). “Severity” in the heatmap indicated the severities of COVID-19, i.e., mild, severe, and critical classifications. As indicated by the heat map, the features that positively correlated with patient classifications included coagulation indices, e.g., PT (Spearman correlation 0.66), APTT (Spearman correlation 0.32), and D-dimer (Spearman correlation 0.72). However, other indices, including PLT (Spearman correlation − 0.27) TC (Spearman correlation − 0.43), HDL (Spearman correlation − 0.61), and LDL (Spearman correlation − 0.36), exhibited a significantly negative correlation with patient classifications. We further applied those data to the normal distribution curve to estimate the relationship of those features with severity (Fig. 4). Unlike the age-severity distribution with the critical group’s mean between the severe group and mild group (Fig. 4), coagulation index-severity distributions, including PT, APTT, and D-dimer, were positively associated with the mild-severe-critical distribution. Other indices, such as TC, HDL, and LDL, were negatively associated (Fig. S2). To explore which indices played an indispensable role, a random forest model was constructed according to patient classifications. The best accuracy of the model is 83.8%, the maximum depth of the tree is 9, and the number of classifiers is 50 (Fig. S2). The model revealed the importance of each feature (Fig. 4, Fig. S2). The most important feature was PT followed by D-dimer. These two features contributed 40% importance to the total. The red dotted line together with the black dotted line separated the features that totaled 90% importance. Taken together, these data suggest the important role of coagulation and hematological indices during the progression of COVID-19.

## Discussion

This study combined 9 autopsy results with the epidemiological and clinical characteristics of 407 COVID-19 patients to explore the dynamic changes in coagulation function profiles during the entire hospitalization. Based on the evaluation of 11 hematological indices on admission to discharge, we found several interesting phenomena that were not previously reported. Of note, those patients received no routine anticoagulant regimen in our observation. These hematological indices, such as PT, APTT, PLT, and D-dimer, exhibited significant changes among different types of patients. Notably, in our study, deceased patients were categorized as critical patients. Mortality among critically ill patients was as high as 48.1%. Moreover, a high level of FIB was noted in the nonsurvivors at days 5–10 before the outcome in our study, which differed from previous reports [5, 18]. Therefore, without further evidence, the high level of FIB should be carefully interpreted with outcome for every patient. In the same period when FIB was higher in the nonsurvivors, the other hemostasis-related indices, such as PLT, PT, and D-dimer, deviated from the normal range, indicating a hypercoagulation state in the nonsurvivors. Of note, PLT was significantly reduced in the critical group and then gradually increased at the late stage of hospitalization (Fig. 2). However, when separating the critical group into subgroups, we found that there were few critical changes in the survivors, and we could reason that the decline in PLT was the result of nonsurvivor thrombocytopenia (Fig. 2**,** Fig. 3). Consistent with a previous study [5], D-dimer levels exhibited two marked peaks during hospitalization in nonsurvivors (Fig. 3), suggesting coagulation activation during thrombosis. Considering the many coagulation-related abnormalities, we also calculated the ISTH DIC score to evaluate the DIC state in all patients along the time axis. Despite the increased FIB level in nonsurvivors, the DIC score showed that the critical patient reached the scoring limit (≥5 according to the ISTH) approximately all the time with no significant difference in its survivor or nonsurvivor subgroup (Fig. 3). This phenomenon of DIC together with the observation of thrombosis from autopsy histological results showed different disturbed hemostatic states among different levels of severity in COVID-19 patients.

Other hemostasis relevant indices include LDH and HDL. Surprisingly, our observation during hospitalization exhibited a significant decrease in these 2 indices in critical patients instead of an increase in the previous report [36]. Given the protective effect of HDL through inhibiting blood vessel aggregation, inflammation, oxidation, endothelial damage and thrombosis in several hematological diseases [37], the low levels of HDL and LDL in our observation in critical patients indicated a disturbed hematological system, which might contribute to disease deterioration. Although GLU was much higher in the critical group and diabetes mellitus was found to be a risk factor for COVID-19 progression, especially for deaths in previous studies and our studies [38], we should still be careful when giving suggestions regarding diabetes and COVID-19 since no direct evidence showed the causality between diabetes and COVID-19 unless more definite conclusions are made through detailed research. Previous studies have demonstrated that several COVID-19 patients exhibit increased concentrations of proinflammatory cytokines, such as tumor necrosis factor-α (TNF-α) and interleukin (IL) [39], especially inducing a cytokine storm that might lead to the activation of the coagulation cascade in severe cases [40]. In addition, diabetes can also affect vascular abnormalities and promote the increased synthesis of glycosylation end products (AGEs) and pro-inflammatory cytokines and oxidative stress to mediate inflammation [41]. Taken together, these results showed that complex CAC progresses in COVID-19 patients with thrombotic complications.

### Limitations

Our study has notable limitations. First, the number of patients included in our study is not large, especially the number of deceased patients. This low number of patients may bias the proportion of comorbidities and other observations. It would be better to include more patients worldwide and among different countries. Second, indices are still not sufficient to evaluate the comprehensive aspects of thrombogenesis given that thrombogenesis is a complex complication. Third, the records started from admission instead of the onset of illness, so part of the coagulation information might be lost.

## Conclusions

In summary, our study provides the full spectrum of coagulation progress with a definite discharge status and shows the existence and dynamic changes of DIC along with this progress. Importantly, we combined autopsy histology and statistical analysis to reveal the significance of hemostasis-relevant indices during thrombosis. These results might help guide therapy and detect prognosis at different levels of COVID-19 infection.

## Supplementary Information

**Additional file 1: Table S1.** Statistical test results related to Table 2.

**Additional file 2: Table S2.** Demographic and clinical features between survivors and nonsurvivors of 81 critically ill patients with COVID-19.

**Additional file 3: Table S3.** Median, interquartile and statistical test results across different hospitalization days among mild, severe, critical patients related to Fig. 2.

**Additional file 4: Table S4.** Laboratory indices of maximum values in individual patients with mild, severe, critical syndromes during the hospital stay.

**Additional file 5: Table S5.** Statistical test results related to Table S4.

**Additional file 6: Table S6.** Median, interquartile and statistical test results across different hospitalization days between survivors and nonsurvivors on different days before outcome related to Fig. 3.

**Additional file 7: Table S7.** laboratory indices among survivors and nonsurvivors of 81 critically ill patients with COVID-19.

**Additional file 8: Figure S1.** Clinical information and outcomes. STROBE diagram of the observation study of COVID-19-infected patients. A total of 408 patients with COVID-19 were enrolled for the analysis. A total of 253, 73, and 81 patients were categorized as mildly, severely, and critically ill patients, respectively.

**Additional file 9: Figure S2.** Informatic analysis of mild, severe and critical patients with COVID-19. Histograms depicting the distribution among features, i.e., age (A), APTT (B), GLU (C), TC (D), and HDL (E), within three patient categories. The abscissa represents the current feature value, and the ordinate represents the probability with the current feature value. The blue bar is the histogram of all samples, indicating the distribution of data. The three fitting curves are black, purple, and red, corresponding to the three patient categories: mild, severe, and critical. (F) The parameters of the random forest model.
