# Supplementary figures and images for: Autopsy and statistical evidence of disturbed hemostasis progress in COVID-19: medical records from 407 patients
Source: Thromb J. 2021 Feb 10;19:8. doi: 10.1186/s12959-020-00256-5 (PMC7873516; doi:10.1186/s12959-020-00256-5)

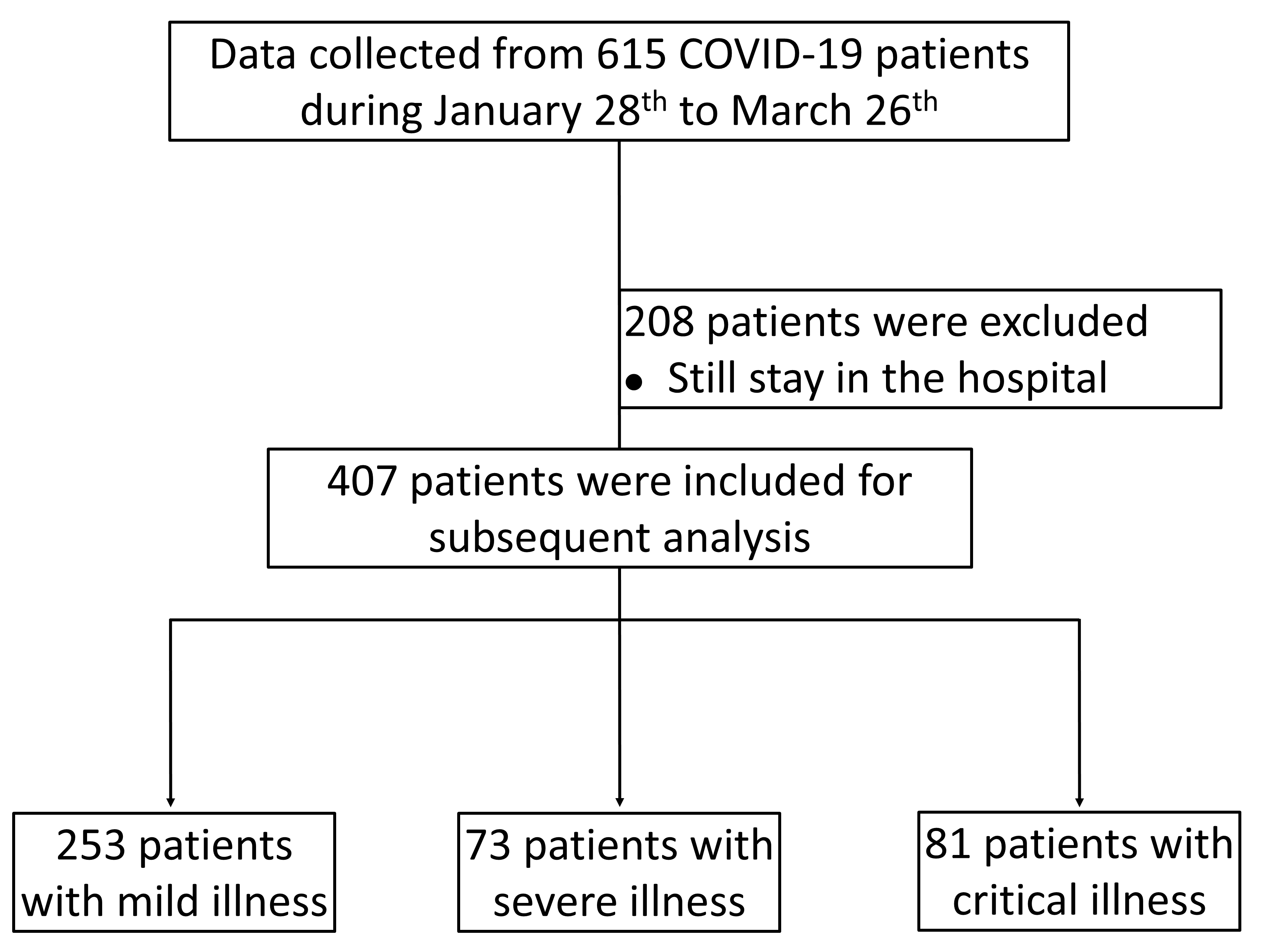

Supplement: Supplementary file 8 — Additional file 8: Figure S1. Clinical information and outcomes. STROBE diagram of the observation study of COVID-19-infected patients. A total of 408 patients with COVID-19 were enrolled for the analysis. A total of 253, 73, and 81 patients were categorized as mildly, severely, and critically ill patients, respectively. [file 12959_2020_256_MOESM8_ESM.tif]

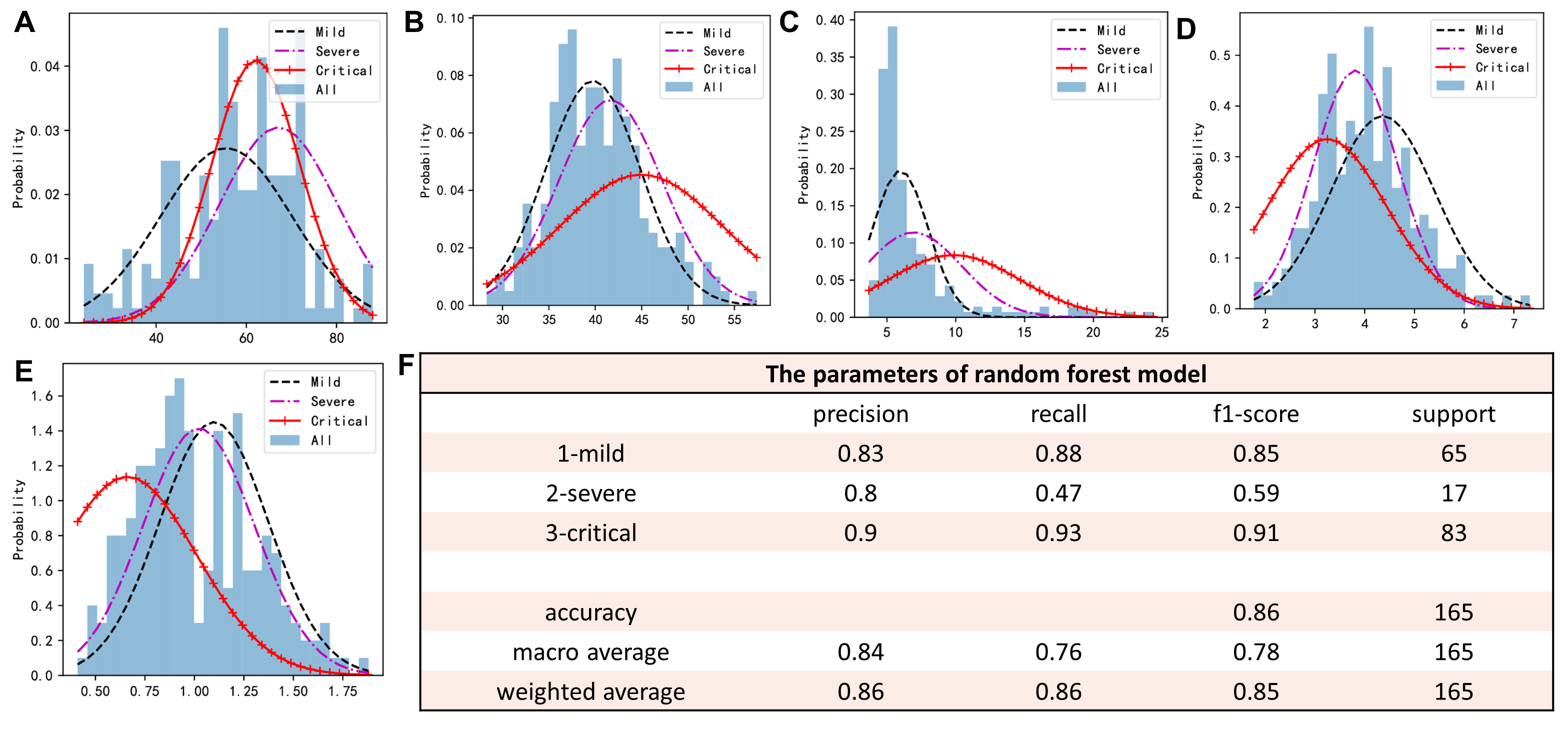

Supplement: Supplementary file 9 — Additional file 9: Figure S2. Informatic analysis of mild, severe and critical patients with COVID-19. Histograms depicting the distribution among features, i.e., age (A), APTT (B), GLU (C), TC (D), and HDL (E), within three patient categories. The abscissa represents the current feature value, and the ordinate represents the probability with the current feature value. The blue bar is the histogram of all samples, indicating the distribution of data. The three fitting curves are black, purple, and red, corresponding to the three patient categories: mild, severe, and critical. (F) The parameters of the random forest model. [file 12959_2020_256_MOESM9_ESM.tif]
